# Scale up of a multi-strategic intervention to increase implementation of a school healthy canteen policy: findings of an intervention trial

**DOI:** 10.1186/s12889-018-5786-x

**Published:** 2018-07-11

**Authors:** Kathryn L. Reilly, Nicole Nathan, John Wiggers, Sze Lin Yoong, Luke Wolfenden

**Affiliations:** 1 School of Medicine and Public Health, University of Newcastle, Callaghan, NSW 2308 Australia; 2 Hunter Medical Research Institute, Newcastle, NSW 2300 Australia; 3 Priority Research Centre for Health Behaviour, University of Newcastle, Newcastle, NSW 2308 Australia; 4 Hunter New England Population Health, Locked Bag 10, Wallsend, NSW 2287 Australia

**Keywords:** Implementation, Schools, Nutrition, Policy, Canteen, Scale

## Abstract

**Background:**

Implementation interventions delivered in schools to improve food provision have been found to improve student diet and reduce child obesity risk. If the health benefits of food availability policies are to be realised, interventions that are effective need to be implemented at scale, across an entire population of schools. This study aims to assess the potential effectiveness of an intervention in increasing the implementation, at scale, of a healthy canteen policy by Australian primary schools.

**Methods:**

A non-controlled before and after study was conducted in primary schools located in the Hunter New England region of New South Wales, Australia. Schools received a multi-component intervention adapted from a previous efficacious and cost-effective randomised control trial. The primary trial outcome was the proportion of canteen menus compliant with the state healthy canteen policy, assessed via menu audit at baseline and follow-up by dietitians. Secondary outcomes included policy reach and adoption and maintenance policy implementation.

**Results:**

Of the 173 schools eligible for inclusion in the trial, 168 provided menus at baseline and 157 menus were collected at follow-up. At follow-up, multiple imputation analysis found 35% (55/157) of schools compared to 17% (29/168) at baseline (OR = 2.8 (1.6–4.7), *p* = < 0.001) had menus compliant with the state healthy canteen policy. As an assessment of the impact of the intervention on policy reach, canteen manager and principal knowledge of the policy increased from 64% (*n* = 76) and 38% (*n* = 44) respectively at baseline to 69% (*n* = 89) and 60% (*n* = 70) at follow-up (*p* = 0.393, *p* = 0.026). Adoption of the policy increased from 80% (*n* = 93) at baseline to 90% (*n* = 104) at follow-up (*p* = 0.005) for principals, and from 86% (*n* = 105) to 96% (*n* = 124) (*p* = 0.0001) for canteen managers. Multiple imputation analysis showed intervention effects were maintained six-months post intervention (33% of menus compliant OR = 2.6 (1.5–4.5), *p* = < 0.001 compared to baseline).

**Conclusions:**

This study found school canteen compliance with a healthy food policy increased in association with a multi-strategy intervention delivered at scale. The study provides evidence for public health policy makers and practitioners regarding strategies and modes of support required to support improvement in nutrition policy implementation across entire populations of schools.

## Background

Globally in 2013, 24% of boys and 23% of girls were classified as overweight or obese (ages 2–19 years) [1]. Childhood overweight and obesity is a predictor of adult obesity, which is associated with chronic diseases including cardiovascular disease, diabetes and some cancers [2–5]. As a result, the economic costs of overweight and obesity to individuals and society are considerable [6].

Schools are recommended by the World Health Organisation (WHO) as a critical setting to improve public health nutrition and to reduce the risk of unhealthy weight gain in childhood [7]. Given this, policies have been introduced in the school setting in a number of jurisdictions internationally that support the provision of food in line with national dietary guidelines [8, 9]. For example, in the United Kingdom, the Department of Education in 2015 mandated the ‘School Food Plan’; a set of standards which requires schools to provide children access to healthy, nutritious meals at school [10]. Similarly, in Australia, where children can purchase foods and drinks over the counter from a canteen or tuckshop [11], all states and territories have introduced mandatory healthy canteen policies that promote the purchase of healthy foods and restrict the sale of less healthy foods [9].

In 2005 in the state of New South Wales (NSW), Australia, the Fresh Tastes @ School Healthy Canteen Strategy was developed and mandated by the Department of Education for government schools to promote the availability of healthy food options in school canteens and limit the sale of foods with poor nutritional value [12]. The policy involves the use of a ‘traffic light’ system that categorizes canteen menu items based on their nutritional value. Schools are required to have a canteen menu dominated (> 50%) by ‘green’ (healthier) food options, to limit the availability of ‘amber’ foods and drinks (less healthy) and to restrict the sale of ‘red’ (poor nutritional value) items [12]. A ‘Sugar Sweetened Drink Ban’ restricting the sales of all sugar sweetened drinks was also introduced in NSW in 2007 [12]. Local population health services are responsible for providing policy implementation support to schools as part of usual service delivery practice.

Despite the existence of school nutrition policies and guidelines, international research suggests that most schools fail to implement them [13, 14]. For example, results of the 2014 School Health Policies and Practices Study in the United States found that 95% of secondary schools sold sugar sweetened beverages and the percentage of schools where fruit and vegetables were available for purchase was approximately 6% [15]. Similarly, a 2007 survey of 50 schools in New Zealand found 84% of schools sold foods in contravention of the guidelines and only 48% had fruit on the menu [16]. Likewise, Woods et al. (2014) analysed a total of 263 school menus from all states and territories in Australia and found variable compliance with state policies from as low as 5 to 62% [9] indicating a clear deficit between the existence of school nutrition policy and its implementation. Hills and colleagues (2015) assessed canteen menus in an Australian region over time (2007–2010) and found little improvement in policy adherence [17].

Despite the importance of implementing school nutrition policies, few trials have investigated the effectiveness of strategies that support the scaled-up implementation by schools of nutrition initiatives broadly, and of policies governing the availability of food in school canteens and food service settings specifically. Three randomised controlled trials of varying implementation support intensity have recently been conducted in NSW, Australia, to enhance the implementation of the state-based school healthy canteen policy [18–20]. Specifically, relative to control groups, schools receiving a ‘high’ intensity implementation support consisting of bi-monthly school visits, executive support, consensus processes, staff training, provision of tools and resources, academic detailing, recognition, performance monitoring and feedback and marketing strategies reported an absolute improvement in menus adherent to the state-based school healthy canteen policy of 56% (RR = 14.41; 95% CI: 2.08, 99.97, *p* = < 0.001) [21]. Similarly those receiving a ‘medium’ intensity implementation support involving similar strategies, in conjunction with a less expensive mode of on-going support (text messaging as oppose to school onsite-visits) reported an absolute improvement of 27% (RR = 4.29; 95% CI: 1.04, 17.68, *p* = 0.02) [21]. The implementation support strategies tested in both of these trials were shown to be cost-effective [21].

To our knowledge these controlled trials form part of the very limited evidence base of strategies to improve implementation of healthy canteen policies globally [22]. While the three trials provide evidence of the effectiveness and cost-effectiveness of implementation strategies and modalities that support policy implementation, they were conducted in relatively small numbers of schools (approximately 35 schools per intervention group). If the health benefits of interventions are to be realised, interventions need to be effective when implemented at scale, across an entire population of schools [23, 24]. Scaling up of a proven intervention from small well controlled and defined research studies into population wide implementation presents unique challenges related to workforce capacity, infrastructure limitations, and catering for a greater diversity of implementation contexts, including differences in geographic or socio-economic characteristics [25].

Research suggests program implementation and effectiveness may attenuate as programs are attempted to be implemented, in real world contexts, as scaling up effective interventions has been associated with a reduction in the impact of implementation support [26, 27] For example, a randomised trial in Australian childcare services tested an intervention to support implementation of practices recommended to improve child physical activity in 20 services [28]. In the 10 services receiving implementation support, substantial improvements of over 40% in most instances in practice implementation were evident [28]. A large scale quasi experimental trial, assessed the impact of attempts to implement such practices, at scale, in the same region across 300 childcare services [26]. The implementation strategy was modified slightly to enable delivery at scale, including the removal of on-site visits however, the implementation support was largely ineffective with no significant improvements in eight of the 11 practices targeted [26].

At present, there are no reported trials of strategies to support the implementation of school healthy canteen policies at-scale. To address this evidence gap, the aim of this study is to assess the potential effectiveness of an intervention in increasing the implementation, at scale, of a healthy canteen policy by Australian primary schools.

## Methods

### Design and setting

A non-controlled before and after study, which is acknowledged as an appropriate design for interventions at this scale [29] was conducted in primary schools located in the Hunter New England region of NSW, Australia. The Hunter New England region covers a large geographical region (more than 130,000km^2^) and consists of a socioeconomically and demographically diverse population of approximately 112,000 children aged 5–12 years [30] and over 400 primary schools.

Approval to conduct the study was obtained from the Hunter New England Human Research Ethics Committee (no. 06/07/26/4.04), The University of Newcastle Human Research Ethics Committee (Approval Number H-2008-0343) as well as the NSW Department of Education and the relevant Catholic Schools Offices.

### Sample

All primary schools (serving children aged 5–12 years) (*n* = 338) in the study region identified via health service record as having an operational canteen were eligible to participate. Schools were ineligible if they catered for secondary students (children aged 13–18 years old), were special purpose schools, that is, catering for students with special needs, juvenile justice or hospitalised, or had already participated in other trials by the research team [18–20, 31].

### Recruitment

Principals of all eligible schools were sent an information letter inviting them to participate in the study. Two weeks following receipt of the invitation, principals were telephoned by a trained research assistant, who confirmed school eligibility, and sought their consent to complete a 20-min Computer-Assisted Telephone Interview regarding school canteen characteristics and policy knowledge and adoption. The interview was conducted during February–April 2016. At the conclusion of interview principal consent was sought to forward an information letter to the school canteen manager inviting them to attend training workshops and to receive support to implement the policy.

### Multi-component implementation intervention

#### Theoretical framework

Rogers’ Diffusion of Innovation Theory a framework for designing health prevention innovations at scale was chosen to guide the development of the intervention [32, 33]. The theory identifies a number of characteristics of an innovation that impact on the rate of adoption by the target population including; the innovation being perceived to have greater advantage over what they are currently doing; be compatible with how they work; be of less complexity; be easily trialled first; and have visible results [32].

#### Intervention to support implementation at scale

To facilitate the implementation of the state healthy canteen policy across the population of schools in the study region, a previous efficacious and cost-effective randomised control trial was replicated [19]. In order to address an identified barrier to policy implementation, that being the classification of menu items according to policy guidelines [34] and to enable implementation support across a large geographical area, an online canteen product database was included as an additional strategy [35]. The intervention was delivered in partnership with the local population health service as part of its usual service delivery practice [36]. The intervention was delivered over a nine-month period (Feb-Oct 2016). The intervention strategies involved the following:

1. Leadership support - An information letter was sent to all eligible school principals and canteen managers providing an overview of the state healthy canteen policy requirements and informing them of an upcoming implementation training workshop and resources available. Principals were sent information regarding the training workshop via email and mail and asked to support and encourage the canteen manager and a parent representative to attend the training workshop and to participate in receiving ongoing support. Securing leadership support has been associated with implementation success [37].
2. Consensus processes - A consensus process involving the canteen manager, canteen staff and/or parent representative was undertaken [38]. A canteen policy implementation action plan was developed. The action plan outlined the school’s goals and key tasks towards implementation of the policy.
3. Education - One-day (5 h) face to face group training workshops were delivered to canteen managers and parent representatives to provide education and skill development [38] in: categorizing menu items according to the policy guidelines; use of a canteen product database and website; financial management of canteens including stock selection, pricing, promotion and operation; and managing volunteers.
4. Tools and resources - Canteen managers received a manual of resources to facilitate implementation [39] of the state healthy canteen policy including: sample canteen policies; planning templates; pricing guides; online product database instructions; guidelines for small schools and self-assessment forms.
5. Provision of implementation support - Schools received at least one contact per school term by a school support officer (trained dietitian) across the intervention period (minimum of four contacts). Contact was made via email, telephone or text messaging with the aim to review implementation progress, prompt action plan delivery and facilitate problem solving [39].
6. Reinforcement - Throughout the intervention period, schools whose canteens were assessed to be compliant with the state policy received a letter of recognition from the research team to acknowledge their positive change [32].
7. Audit and feedback – Audit and feedback has been shown to produce significant practice changes [40, 41] Schools received up to two menu audit and feedback reports regarding canteen progress towards achieving implementation action plan goals (Summer and Winter menus). Canteen menus were collected via school administration personnel and assessed according to the policy criteria. The reports identified menu food and beverage items that were restricted for sale and made suggestions for suitable replacements [42].
8. Canteen product database - a canteen product database was developed and placed on the project website (*Good for Kids. Good for Life* website) [35] to provide access to a range of potential products coded according to the state healthy canteen policy.

### Data collection and measures

School principals were invited to participate in a telephone interview regarding school characteristics and policy knowledge and adoption at baseline (Feb-Apr 2016) and again immediately post intervention (Nov-Dec 2016). Canteen managers who attended the training workshops were invited to complete a pen-paper survey prior to commencing the training workshops (Feb-Apr 2016). Canteen managers who did not attend the training workshops were contacted via telephone and invited to complete the survey via a computerised assisted telephone interview. All canteen managers were contacted immediately post intervention to complete a follow-up telephone interview (Nov-Dec 2016).

#### School and canteen characteristics

Information regarding school size (number of enrolled students), school type (Government, Catholic or Independent) and the locality of the school (school postcode) was collected from school websites and school databases. Canteen characteristics such as days of operation, staffing and management of the canteen were collected through the baseline canteen manager interview.

#### Exposure to other nutrition interventions

During the follow-up telephone interview, canteen managers were asked to report any exposure to and/or involvement in other initiatives to assist with the implementation of the policy.

### Outcomes

Assessment of the trial outcomes of the intervention was informed by the RE-AIM evaluation model [43] and involved four of the RE-AIM domains. Implementation of the policy (compliance) was the primary trial outcome. Measures of Reach, Adoption and Maintenance [44] were identified as secondary. We did not re-assess ‘effectiveness’ of the intervention on dietary outcomes at the level of individual students as that has previously been found to be effective in improving the nutritional quality of foods purchased [20], and the effectiveness of the intervention is supported by a systematic review of experimental research [45].

#### Primary trial outcome - compliance with the ‘Fresh Tastes @ School’ policy

The primary trial outcome was the proportion of canteen menus that were compliant with the state policy [12]: defined as containing no ‘red’ or ‘banned’ menu items and having > 50% ‘green’ menu items. We also report the proportion meeting each of these two criteria separately. Outcome data were collected at baseline and follow-up via audits of canteen menus faxed or emailed to the project team by the school. Menus were audited by a dietitian, trained in menu classification, using a validated Quick Menu Audit tool [46]. The tool consisted of a list of common canteen menu items grouped into categories such as drinks, hot food, frozen dairy treats, snacks, sandwiches and salads. The tool included colour coded classifications and justifications for assumptions made regarding menu item details such as brand and portion size, to categorise menu items as ‘green’, ‘amber’, ‘red’ and ‘banned’ according to the criteria specified by the state policy.

Menu compliance was determined by tallying all items on the menu, and determining the percentage of items that were categorised as either ‘green’, ‘amber’, ‘red’ and ‘banned’.

#### Secondary outcomes

##### Policy reach

As a measure of school exposure to the policy (reach) by assessing awareness of it, principals and canteen managers were asked during telephone interviews, to identify the intent of the state policy. Specifically, principals and canteen managers were asked which one of the following statements they thought was consistent with the policy; “Foods high in saturated fat, salt or excess kilojoules; a) should not be available for regular sale (correct response); b) can be sold regularly but must not comprise more than 10% of items listed on canteen menus; or c) can be sold regularly but schools must have 2 days per term where such foods are not available”.

##### Policy adoption

As a measure of stage of adoption, during the telephone interviews, principals and canteen managers were asked: “Which of the following statements best represents your school’s intent to use the Fresh Tastes @ School guidelines?” Based on the Alberta Nutrition Guideline Outcomes Telephone-Survey Questionnaire [47], respondents were asked to categorise their school according to the five stages of behaviour change; 1. We have not thought about using the Fresh Tastes guidelines in the canteen / Don’t know (pre-contemplation); 2. We are thinking about using the Fresh Tastes guidelines in the canteen (contemplation); 3. We are planning to or have taken some steps to using the guidelines in the canteen (preparation); 4. We are currently using the Fresh Tastes guidelines (action); or 5. We have been using the Fresh Tastes guidelines for more than 6 months (maintenance) [48]. (Table 3).

##### Implementation maintenance

Maintenance of implementation of the policy, was assessed by measuring compliance (primary outcome), six months after the immediate post-intervention outcome follow-up measure.

#### Process evaluation

Project records were used to determine the proportion of schools that: received principal information letters, developed action plans, attended training workshops, received tools and resources, received menu feedback reports, and received on-going support via text messaging or email. Acceptability of the training workshop content was measured through a pen and paper survey conducted at the completion of workshops.

## Analyses

All analyses were conducted using the statistical package SAS 9.3 (SAS Institute Inc., Cary, NC). Descriptive statistics were used to describe the demographic, school and canteen characteristics of the group. The number of enrolled students in each school were used to categorise school size as small (1–159 students), medium (160–450) or large (> 450 students) based on the NSW Department of Education’s classifications of school size [49]. School socio-economic status was based on postcode. Similar to other Australian based implementation studies [18–20], ‘higher socio-economic status’ were those schools ranked in the top 50% of NSW, whilst ‘lower socio-economic’ status was the bottom 50% [50]. School postcode was also used to describe locality; ‘rural’ defined as outer regional, remote and very remote areas, ‘urban’ defined as regional cities and inner regional areas [51].

Pre-post differences were assessed using mixed effects logistic regression models to assess the impact of the intervention on the following compliance outcomes; overall compliance, no ‘red’ items on the menu and greater than 50% ‘green’ items, as per policy requirements [12]. Exploratory chi-square analysis was performed to assess whether there was an association between compliance at follow-up and school characteristics. All analyses were performed on complete case data, where schools provided menus at both baseline and follow-up (primary outcome) or maintenance (secondary outcome). Additionally, analyses employing multiple imputation was performed for schools with missing data at either follow-up or maintenance.

Pearson Chi-square tests were used to measure pre-post differences in the measure of ‘reach’ - proportion of principals and canteen managers who could correctly identify the statement consistent with the policy. For the adoption measure, schools who responded they were in the preparation, action or maintenance stage of change were classified as ‘adopters’ whilst schools in the pre-contemplation and contemplation stages were classified as ‘non-adopters’ [52].

## Results

### Participants and characteristics

Of the 338 schools in the study region identified as having an operational canteen, 173 schools were deemed eligible for participation. Twenty-four schools had secondary students, and 134 had participated in trials conducted by the research team [18–20, 31]. Seven principals reported they had no operational canteen during the baseline telephone interview and were therefore excluded from the study. At baseline 168 (97%) schools provided their menu for assessment and 125 (72%) principals and 122 (71%) canteen managers completed their respective telephone interviews. At follow-up, 157 schools provided their menu for assessment, eight schools reported they had recently closed their canteen and four refused to participate. The proportion of canteen managers and principals who completed the follow-up telephone interviews was 129 (75%) and 115 (66%) respectively.

Table 1 outlines the baseline characteristics of all eligible schools. Small schools (< 160 students) (*p* = 0.002), schools categorised as being in lower socioeconomic regions (*p* = 0.01) and those located in outer regional or remote areas (*p* = 0.04) were more likely to not provide a menu at follow-up.

**Table 1:** Baseline characteristics of eligible schools

Characteristics	Intervention Group *n* = 173 (%)
School Type	
Government	129 (75)
Catholic	40 (23)
Independent	4 (2)
School size	
Small (1–159 students)	77 (45)
Medium (160–450 students)	81 (47)
Large (450+ students)	15 (9)
Urban/Rural region^a^	
Major Cities + Inner Regional	149 (89)
Outer Regional + Remote Australia	19 (11)
Socio-economic index^b^	
Lower Socio-economic areas	102 (61)
Higher Socio-economic areas	64 (39)
Management of canteen	
Parent Representative Groups	105 (86)
Principal / School run	14 (11)
Contracted external food service	1 (1)
Contracted off-site caterer	0(0)
Other	2 (2)
Canteen Staff^c^ (may select more than one option)	
Paid manager / supervisor	39 (32)
Paid assistant(s) /workers /parents	6 (5)
Volunteer manager / supervisor	56 (46)
Volunteer workers / parents	109 (89)
Contractor	0 (0)
Student help	6 (5)
Other	2 (2)
Days of operation	
5 days / week	55 (45)
4 days / week	10 (8)
3 days / week	21 (17)
2 days / week	9 (7)
1 day / week	26 (21)
Less than 1 day / week	1 (1)

^a^5 missing data

^b^7 missing data

^c^Percentages greater than 100 as participants may select more than one response

### Primary trial outcome - compliance with the state policy

As seen in Table 2, 41% (64/157) of schools at follow-up had no ‘red’ or ‘banned’ menu items compared to 24% (41/168) at baseline (*p* = 0.002) and 72% (113/157) had greater than 50% ‘green’ menu items compared to 62% (104/168) at baseline (*p* = 0.043). In terms of overall compliance with the state policy, 35% (55/157) of schools at follow-up compared to 17% (29/168) at baseline (OR = 2.7 (1.6–4.7), *p* = < 0.001) had menus compliant with the state heathy canteen policy. A similar effect was found using multiple imputation for missing data (OR = 2.8 (1.6–4.7), *p* = < 0.001).

**Table 2:** Primary outcome: implementation

	Baseline n (%)	Follow-up n (%)	Complete Case (*n* = 157)		Multiple Imputations (*n* = 168)	
			Odds Ratio (95%CIs)	*p*-value	Odds Ratio (95%CIs)	*p*-value
No red/banned	41 (24)	64 (41)	2.4 (1.4–3.7)	0.001^*^	2.3 (1.4–3.7)	< 0.001^*^
> 50% green	104 (62)	113 (72)	1.7 (1.0–2.9)	0.043^*^	1.7 (1.0–2.8)	0.05
Overall compliance	29 (17)	55 (35)	2.7 (1.6–4.7)	< 0.001^*^	2.8 (1.6–4.7)	< 0.001^*^

*Statistically significant

#### Exploratory analysis

Exploratory analysis of compliance rates at follow-up based on school and canteen characteristics identified government schools as significantly more likely to have menus compliant with the policy than Catholic or Independent schools (*p* = 0.049). There was no other statistically significant difference between characteristics such as school size (*p* = 0.779, geographical location (*p* = 0.428), socio-economic status (*p* = 0.17), canteen management (*p* = 0.115), or days of operation (*p* = 0.761) in terms of compliance at follow-up.

### Secondary outcomes

Policy reach and adoption results are outlined in Table 3. Canteen managers and principals who correctly identified the statement consistent with the policy increased from 64% (*n* = 76) and 54% (*n* = 63) respectively at baseline to 69% (89) and 68% (*n* = 79) respectively at follow-up (*p* = 0.38, *p* = 0.034). The proportion of canteen managers who completed the telephone interview classified as ‘adopters’ increased from 86% (*n* = 105) at baseline to 97% (*n* = 124) at follow-up (*p* = < 0.0001). Likewise, the proportion of principals who were classified as ‘adopters’ increased from 80% (*n* = 93) at baseline to 90% (*n* = 104) at follow-up (*p* = 0.0001). Similar effects were seen with multiple imputation analysis for both policy reach and adoption (Table 3).

**Table 3:** Secondary outcomes: reach and adoption

RE-AIM domain	Canteen Managers Baseline n (%)	Canteen Managers Follow-up n (%)	Complete Case (*n* = 99^a^, 100^b^)	Multiple Imputations (*n* = 122)	Principals Baseline n (%)	Principals Follow-up n (%)	Complete Case (*n* = 88)	Multiple Imputations (*n* = 125)
			*p*-value	*p*-value			*p*-value	*p*-value
Reach	76 (64)	89 (69)	0.38	0.41	63 (54)	79 (68)	0.034^*^	0.036^*^
Adoption	105 (86)	124 (97)	0.0001^*^	< 0.0001^*^	93 (80)	104 (90)	0.0001^*^	< 0.001^*^

^*^Statistically significant, ^a^ = Reach Outcome, ^b^ = Adoption Outcome

Of the 148 schools who provided menus six-months post intervention (implementation maintenance), 33% (*n* = 48, OR = 2.4 (1.4–4.0), *p* = 0.001 compared to baseline) had menus that were compliant with the state policy an effect that remained significant following multiple imputation for missing data (OR = 2.6 (1.5–4.5), *p* = < 0.001) (Table 4).

**Table 4:** Secondary outcomes: maintenance

	Baseline n (%)	6-months maintenance n (%)	Complete Case (*n* = 148)		Multiple Imputations (n = 168)	
			Odds Ratio (95%CIs)	*p*-value	Odds Ratio (95%CIs)	*p*-value
No red/banned	41 (24)	57 (39)	2.0 (1.2–3.3)	0.007^*^	2.1 (1.3–3.5)	0.003^*^
> 50% green	104 (62)	100 (68)	1.3 (0.8–2.1)	0.29	1.3 (0.8–2.2)	0.26
Overall compliance	29 (17)	49 (33)	2.4 (1.4–4.2)	0.001^*^	2.6 (1.5–4.5)	< 0.001^*^

^*^Statistically significant

### Process evaluation

Table 5 shows the proportion of schools who received each of the implementation strategies. All schools were mailed the principal information letter, sent text messages or emails as part of on-going support, received tools and resources at workshops or mailed to the school, provided with product database information and mailed at least 1 menu feedback report. Almost half (49%) of schools developed an action plan, half (50%) attended the training workshops and 75% supplied a second menu for review and hence received a second menu feedback report.

**Table 5:** Number of schools receiving implementation strategies

Implementation Strategy	Number of schools in sample *n* = 157 n (%)
Principal Information Letter (leadership and buy-in)	157 (100)
Developed action plan (consensus process)	77 (49)
Attended training workshop (education)	79 (50)
Tools and Resources	157 (100)
Text messages or emails (on-going support)	157 (100)
Received 1 menu report (audit and feedback)	157 (100)
Received 2 menu reports (audit and feedback)	117 (75)
Product database	157 (100)

### Exposure to other nutrition interventions

Canteen managers from 22 schools reported receiving support to assist in implementation of the policy beyond that provided by the trial. Nine of these schools reported receiving educational and promotional material from a multi-faceted program to promote the consumption of fresh fruit and vegetables amongst school-aged children. Three schools reported liaising with other canteen managers, three had gained information from manufacturers or suppliers and the remaining seven schools listed unspecific support such as receiving ‘brochures’ and ‘leaflets’.

## Discussion

This is the first study to assess the potential effectiveness of an intervention to support implementation at scale, by 173 schools, of a healthy canteen policy in Australian primary schools. The findings suggest that a multi-strategy intervention involving leadership, consensus processes, education, resources, audit and feedback, and on-going support in the form of text messages/emails may improve schools’ implementation of a healthy school canteen policy at-scale. The study makes a novel contribution to a currently sparse research landscape in the school setting regarding implementation at-scale [24] and provides evidence to support improvement in nutrition policy implementation across populations of schools.

The high level of participation in this study (91%, 157/173) is consistent with that required by scale-up programs to reach a large proportion of the target population in order to have a public health impact [53]. The size of the change in compliance in this study (18%) is similar to the effect sizes in other school based obesity prevention interventions designed to support large numbers of schools’ implementation of health promotion programs (13–45%) [54–57]. The observed change in compliance in this study (18%) is, however, lower than the effects achieved in the randomised control trial from which the study was adapted (29% effect size) [19], a finding that is consistent with previous pragmatic studies [26, 27]. Gottfredson et al. (2015) suggests that adaptations or differences in population characteristics may reduce the effects of interventions when delivered at-scale.

Logistical challenges of expanding implementation into larger and more rural geographic areas appeared to have reduced exposure of the schools to the implementation support provided. For example, just 50% of schools (*n* = 79) attended training and 49% of schools developed an action plan (*n* = 77) compared with 93% (26/28) [19] for both in the original trial. Such findings may be due to the greater distances required for school staff to attend training in this trial compared to the original trial. Further research into ways to extend the reach of strategies such as workshops to rural and remote regions, including the possibility of online training [58], may be warranted.

Whilst the policy is strongly endorsed by the Catholic Schools sector in the region and the Association of Independent Schools, it is mandated for government schools only. Government schools were more likely to have menus compliant with the state healthy eating policy (*p* = 0.049) suggesting a positive relationship between a mandatory policy and implementation. There were no other differences in compliance and school characteristics such as location, size or socioeconomic status, indicating that the intervention is effective across a diverse population of schools. Such findings suggest that the policy implementation approach may not further exacerbate existing nutrition inequalities among these groups.

The observed small increases in knowledge of the policy by canteen managers (5%, *p* = 0.393) found in this study is unsurprising as the policy was first launched over 10 years ago. Although baseline levels of ‘adoption’ were similarly high, there was a small but significant shift in schools’ adoption of the policy for both canteen managers and principals. The proportion of schools in which an intervention effect was maintained (determined at 6-months post intervention menu audit) (33%, *n* = 49) was similar to that at immediate post-intervention follow-up (35%, *n* = 55). As previous research has shown that policy implementation improves student diet [20, 45, 59], the findings demonstrates the potential contribution the implementation support strategy can make in achieving public health nutrition enhancements.

Limitations of the study include its non-controlled study design. Whilst the lack of a control group precludes causal inference that the observed changes over time were the result of the intervention, policy implementation over the past decade has remained stable [17] and steps were taken to assess contamination such as any exposure to and/or involvement in other initiatives to assist with implementation of the policy. A further limitation is possible selection bias, as schools that chose to take part in the intervention may be different from those schools that did not [60]. It is not known whether differences existed, for example, in canteen managers’ self-motivation and/or support from principals in study participants compared to non-participants.

## Conclusion

Despite the introduction of healthy eating policies in schools in many countries their implementation across the population of schools has been limited. Few trials have investigated the effectiveness of strategies designed to increase schools’ implementation of such policies and this study is the first to do so at scale. The study provides novel information for public health policy makers and practitioners regarding strategies and modes of support required to facilitate the implementation of nutrition policies and guidelines broadly and healthy canteen policies specifically across entire jurisdictions.

## Abbreviations

CIs: Confidence intervals
NSW: New South Wales
OR: Odds ratio
WHO: World Health Organisation
